# Large Transverse Piezoelectricity in Highly (001)-Oriented PZT Thick Films on Titanium Substrates

**DOI:** 10.3390/ma19112396

**Published:** 2026-06-04

**Authors:** Zefeng Guo, Jun Ouyang, Shijing Chen, Zhenyan Liang, Hongbo Cheng

**Affiliations:** 1 School of Chemistry and Chemical Engineering, Qilu University of Technology (Shandong Academy of Sciences), Jinan 250353, China; 10431231056@stu.qlu.edu.cn (Z.G.); liangzy@qlu.edu.cn (Z.L.); 2 Department of Physics, Seton Hall University, South Orange, NJ 07079, USA; 3 Hunan Provincial Key Laboratory of Thin Film Materials and Devices, School of Materials Science and Engineering, Xiangtan University, Xiangtan 411105, China; 202321551583@smail.xtu.edu.cn

**Keywords:** PZT film, titanium, (001) orientation, transverse piezoelectric coefficient/*e*_31,*f*_, RF magnetron sputtering, rapid thermal processing (RTP), piezo-MEMS

## Abstract

**Highlights:**

1. Highly (001)-textured PZT thick films were successfully integrated on reactive Ti substrates.
2. A conductive LaNiO_3_ buffer enables simultaneous texture control and interfacial stability.
3. The PZT films exhibit superior piezoelectric performance with a high *e*_31,*f*_ (~−6.7 C/m^2^).
4. This work provides a feasible route for flexible piezo-MEMS on metallic substrates.

**What are the main findings?**

- (001)-oriented and dense PZT thick films (~1.27 μm) were fabricated on Ti substrates by 400 °C RF sputtering +640 °C rapid thermal processing.
- The LaNiO_3_ buffer layer reduces the perovskite nucleation barrier and suppresses Ti substrate oxidation and interfacial diffusion/reaction.
- The PZT film shows excellent ferroelectric and dielectric properties and a high *e*_31,*f*_ of ~−6.7 C/m^2^, outperforming reported Ti-based piezoelectric films.

**What are the implications of the main findings?**

- This work resolves the thermal budget conflict between high PZT crystallinity and device stability on reactive Ti substrates.
- A scalable and cost-effective strategy is provided for the integration of high-performance PZT films for flexible piezo-MEMS devices.

**Abstract:**

Integration of lead zirconate titanate (PZT) films on metallic substrates is important for flexible piezoelectric devices, but achieving highly textured crystallinity without detrimental interfacial diffusion or oxidation remains challenging. In this work, PZT thick films (~1.3 μm) were deposited on titanium substrates using radio-frequency magnetron sputtering at 400 °C followed by rapid thermal processing at 640 °C for 2.5 min. A conductive LaNiO_3_ buffer layer was introduced to promote the nucleation of the perovskite phase and suppress interfacial degradation. The resulting PZT films on the LNO/Pt/Ti substrates exhibit a strong (001) preferred orientation and a dense microstructure. The films show a large remnant polarization *P*_r_ of ~61 μC cm^−2^ and a low coercive field *E*_c_ of ~56 kV cm^−1^ at 60 V, together with a dielectric constant *ε_r_* of ~1350–1612 and a dielectric loss tanδ ≤ 0.06 in the frequency range of 1 kHz to 1 MHz. Patterned Pt/PZT/LNO/Pt/Ti cantilevers yield a transverse piezoelectric coefficient *e*_31,*f*_ of ~−6.7 C/m^2^, significantly outperforming reported piezoelectric films deposited on Ti. These results demonstrate that controlled nucleation and rapid thermal crystallization enable highly textured PZT films on reactive metallic substrates, providing a viable route for flexible piezoelectric MEMS devices.

## 1. Introduction

Lead zirconate titanate (PZT) films, particularly those with morphotropic phase boundary (MPB) compositions [1], have established themselves as the cornerstone of piezoelectric micro-electro-mechanical-systems (Piezo-MEMS) due to their exceptional electromechanical properties. While silicon-based PZT film devices have been extensively investigated [2,3,4,5], the intrinsic brittleness of silicon limits their use in emerging flexible electronic systems such as wearable sensors, flexible actuators and vibrational energy harvesters. In contrast, metallic foils have recently attracted increasing attention as substrates for flexible electronics because of their mechanical ductility, durability, and relatively low cost. Among various metallic substrates, titanium (Ti) is particularly attractive for the integration of PZT films. One important advantage is the close match between the coefficient of thermal expansion (CTE) of Ti (~8.6 × 10^−6^ °C^−1^) and that of PZT (~8.0 × 10^−6^ °C^−1^) [6], which helps reduce thermally induced stresses that may otherwise degrade film integrity or cause delamination during processing. In addition, titanium exhibits excellent corrosion resistance, high toughness, and good manufacturability, making it a promising platform for flexible functional thin-film devices.

Despite these advantages, the integration of high-quality PZT films on Ti substrates remains challenging. Previous investigations via common deposition methods, like sputtering or sol–gel [7,8,9], have consistently shown that achieving a pure perovskite phase with an epitaxial quality or a high texture in a PZT film requires prolonged exposure to a high processing temperature (typically exceeding 500 °C) in an O_2_-rich atmosphere (to suppress the formation of oxygen vacancies). In such a chemical environment, the highly reactive Ti substrate readily undergoes interfacial diffusion and severe oxidation, resulting in the formation of a low-permittivity interfacial oxide layer [10,11,12], or the decomposition of PZT into a non-ferroelectric pyrochlore phase [10]. Both effects significantly degrade the electrical performance of the PZT film [10,11]. Consequently, reducing the thermal budget during film processing is essential for preserving interfacial stability while enabling high-quality PZT growth on Ti substrates.

A two-step processing strategy combining low-temperature deposition with rapid thermal processing (RTP) has been proposed to address this challenge. In this approach, PZT is first deposited at a relatively low temperature and subsequently crystallized through a rapid high-temperature annealing step, thereby minimizing the thermal exposure of the Ti substrate. However, an additional challenge arises in achieving highly desirable (001) crystallographic texture in PZT films deposited on metallic substrates. The large lattice mismatch between (001)-oriented PZT and conventional metallic electrodes such as Pt or Ti typically results in randomly oriented polycrystalline films [6,10], which exhibit reduced piezoelectric performance. Introducing a suitable buffer layer that provides a crystallographic template for the nucleation and growth of (001)-oriented PZT is therefore critical [13,14].

In this work, we demonstrate an optimized two-step fabrication route for producing highly (001)-textured PZT thick films on titanium substrates. The process combines RF magnetron sputtering at 400 °C with rapid thermal processing, along with the introduction of a conductive LaNiO_3_ (LNO) buffer layer. The LNO layer serves as a structural template that promotes the nucleation and growth of perovskite PZT while mitigating the lattice mismatch with the underlying substrate [15,16,17]. In addition, it acts synergistically with the RTP process to suppress interfacial diffusion. As a result, the PZT films grown on the LNO/Pt/Ti substrates exhibit a strong (001) preferred orientation and a dense microstructure, yielding a high remanent polarization *P*_r_ of ~61 μC cm^−2^ and a large transverse piezoelectric coefficient *e*_31,*f*_ of −6.7 C/m^2^. These results demonstrate that controlled interfacial nucleation combined with rapid thermal crystallization enables the formation of highly textured PZT films on reactive metallic substrates.

## 2. Materials and Methods

### 2.1. Film Fabrication

In this study, PZT films with a Zr/Ti atomic ratio of 52/48 were fabricated on LaNiO_3_ (LNO) buffered Pt/Ti substrates. Titanium substrates (TA1 grade, >99.5% purity, wool-wheel polished, 20 × 10 × 0.5 mm) with a Young’s modulus of 104 GPa [18] were used. The PZT/LNO/Pt/Ti thin film heterostructures were prepared in a multi-target magnetron sputtering system equipped with 3-inch sputtering guns, and a base pressure of 2.0 × 10^−4^ Pa was achieved prior to film deposition. The ceramic PZT target (99.9% purity) was purchased from Hefei Kejing Materials Technology Co., Ltd. (Hefei, China), with a 20 mol% excess PbO to compensate for the volatilization loss of lead during target sintering and film processing. A chemically stoichiometric LNO ceramic target (99.9% purity) was supplied by Hefei Anjing Crystal Material Co., Ltd. (Hefei, China), while the Pt and Ti metallic targets (99.99% purity) were purchased from ZhongNuo Advanced Material Technology Co., Ltd., (Beijing, China). A Pt bottom electrode (~300 nm), with a Ti adhesion layer (~10 nm) was first sputtered onto the Ti substrate at 300 °C in pure Ar at a pressure of 0.3 Pa. The sputtering power for both Pt and Ti was 55 W. Secondly, a LNO buffer layer (~120 nm thick) was deposited at 400 °C with a RF power of 100 W from its ceramic target, in an Ar/O_2_ mixed atmosphere (4:1 flow ratio) at a pressure of 0.3 Pa. Finally, a PZT thick film (~1.3 μm) was deposited onto the LNO layer from its ceramic target, at the same temperature of 400 °C and in the same Ar/O_2_ atmosphere, but at a higher sputtering pressure of 1.2 Pa. The detailed deposition parameters are summarized in Table 1.

The as-deposited films were annealed via rapid thermal processing (RTP) in an oxygen atmosphere (1.2 L/min fixed flow rate), using a RTP-300 furnace purchased from the Beijing East-Star Applied Physics Research Institute (Beijing, China). The RTP process involved ramping from room temperature to 640 °C at a rate of 2.5 °C/s, followed by a holding time of 2.5 min. The annealed films were then cooled down to room temperature by switching off the RTP furnace.

### 2.2. Characterization

The crystallographic characteristics, including the phase structure, crystalline orientation and quality of the PZT films, were investigated using X-ray diffraction (XRD) with a SmartLab 9 kW diffractometer (Rigaku, Tokyo, Japan) equipped with a D/teX Ultra 250 1D silicon strip detector. All XRD tests were performed in the conventional *θ–2θ* mode (no grazing incidence mode was adopted). For surface morphology analysis, atomic force microscopy (AFM) was carried out using an AFM 100Plus microscope (Hitachi, Tokyo, Japan). The surface morphology analysis of the obtained AFM images was performed using the bundled software *AFM100 Offline* (version 7.10C2). A silicon cantilever probe (Model: SI-DF40P2) was used for AFM characterization, with manufacturer-provided specifications including a resonant frequency of 276 kHz, a force constant of 24 N/m, aluminum coating, and a standard pyramidal tip shape. Cross-sectional nanoscale structural and chemical composition analyses were performed via transmission electron microscopy (TEM) using a Talos F200X electron microscope (Thermo Fisher Scientific, Waltham, MA, USA) operated at an accelerating voltage of 200 kV, equipped with a Super-X type EDS detector. The elemental mapping was analyzed using the associated software (Velox 3.4) of the instrument. For electrical measurements, Au top electrodes (200 μm in diameter) were deposited on the PZT film surface via DC sputtering through a shadow mask in a SBC-12 vacuum sputter (KYKY, Beijing, China). The dielectric properties were assessed using a TH2838H precision LCR meter (Tonghui, Changzhou, China). The leakage current density, polarization–electric field (*P-E*) hysteresis loops, and the corresponding switching currents were measured using a MultiferroicTM ferroelectric tester (Radiant Technology, Lewis Center, OH, USA).

The transverse piezoelectric coefficients (*e*_31,*f*_) were determined by measuring the tip displacements of a PZT film cantilever. A Pt/PZT/LNO/Pt/Ti heterostructure, with the Pt top electrode deposited via RF magnetron sputtering at room temperature, was diced into cantilever beams with dimensions of 20 mm (length) × 3 mm (width) × 0.5 mm (thickness). The Pt top electrode was connected to a function generator via a fine gold wire, through which a sinusoidal ac voltage was applied to actuate the cantilever. The resulting tip displacements (δ) of the PZT film cantilever were measured using a Laser Doppler Vibrometer (LDV) comprising an OFV-505 sensor head and an OFV-5000 controller (Polytec Inc., Mooresville, NC, USA). Detailed information regarding the fabrication and testing procedures of the piezoelectric film cantilevers can be found in a previous report [16].

## 3. Results

**Figure 1:**
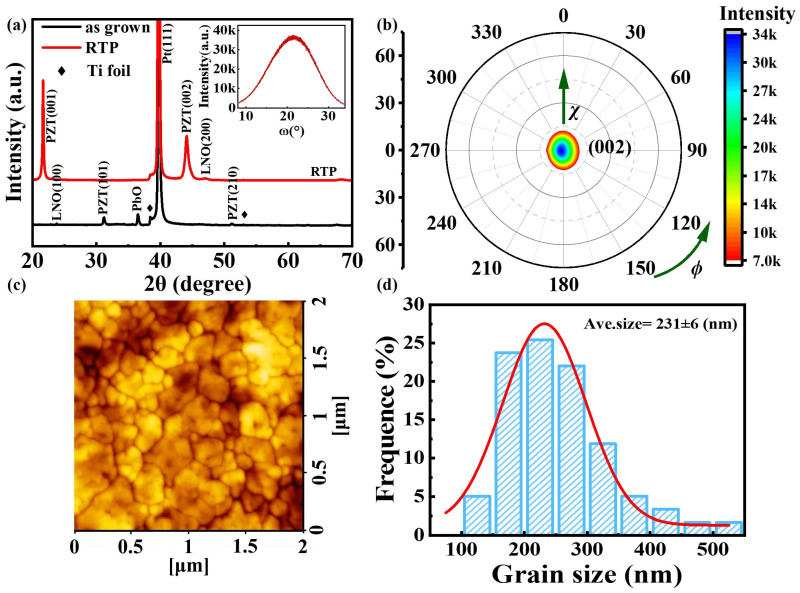
Crystalline phase structure and surface analyses of Pb(Zr_0.52_Ti_0.48_)O_3_ (PZT) films grown on LaNiO_3_ (LNO)-buffered Pt/Ti substrates: (**a**) XRD 2*θ*-scan patterns of the PZT film before and after RTP, with the inset showing the rocking curve of the RTP-treated film in the (002) plane; (**b**) XRD pole figure of the (002) PZT peak, (**c**) AFM surface scan image; and (**d**) histogram of the in-plane grain diameter for the RTP-treated PZT film.

The bedrock of our strategy is to decouple the crystallization step, which can be rapidly achieved at a high temperature with a LNO template, from the PZT film deposition, which demands a long time period and should be carried out at a low temperature. Such a strategy has successfully passivated the Ti and O diffusion, preventing Ti from penetrating into the PZT layer and minimizing the oxidation of the Ti substrate. Figure 1a presents the XRD 2 scan patterns of the PZT film before and after RTP, which unequivocally demonstrate the effectiveness of this two-step approach. The as-deposited film, grown at a low temperature of 400 °C, exhibits some residual PbO crystallites due to suppressed lead volatilization, as well as a poorly crystallized perovskite structure with a (101) preferred orientation. The latter can be attributed to a surface energy-dictated nucleation and growth process at a low temperature, with the (101) plane having the minimal surface energy among perovskite oxides. These observations are consistent with our previous results [19]. In contrast, a dramatic microstructural change occurred after the RTP treatment (@640 °C for just 2.5 min). The film evolved into a pure perovskite phase, evidenced by the disappearance of the PbO peak. More importantly, it displayed a highly crystalline (001)-texture. This crystalline reorientation is a direct consequence of heterostructure engineering. Firstly, a (100)-oriented nucleation and growth was promoted in the as-grown and RTP-annealed LNO film [20,21]. Secondly, with a pseudocubic perovskite structure and a close lattice match to (001) PZT, the (100)-oriented LNO film acted as a crystallographic template [16,21], providing a thermodynamically favorable pathway for the (001)-oriented growth of the PZT film, especially during the high-temperature recrystallization process driven by RTP. The formation of this (001) texture is essential, as this crystallographic orientation aligns with the spontaneous polarization axis in tetragonal PZT, thereby ensuring superior ferroelectric and piezoelectric performance [22,23].

To evaluate the crystalline quality of the resulting PZT film, an XRD rocking curve was acquired for the (002) PZT peak (inset of Figure 1a). The symmetric peak with a maximum count exceeding 30k and a full-width at half-maximum (FWHM) of approximately 6°, confirms a good (001)-textured crystallinity. Furthermore, an XRD pole figure for the (002) PZT peak, shown in Figure 1b, provides a 3D rendering of the (00*l*) texture. The diffraction intensity is highly concentrated near the center of the pole figure (χ ranges from 0° to ~12°) with no other detectable peaks, indicating that the c-axes of the PZT grains in the film are either aligned with or within a small angle to the film normal. An AFM image showing the film’s surface morphology is presented in Figure 1c, revealing a dense film surface with in-plane equiaxial grains. The film exhibits a low root-mean-square (RMS) roughness of ~7.7 nm, an arithmetical mean deviation (Sa) of ~6.2 nm, and a maximum height (Sz/Smax) of ~56.4 nm, and an average in-plane grain diameter of ~231 ± 6 nm (Figure 1d). The average grain size was calculated using the *Nano Measurer* 1.2 software over a 5 × 5 μm^2^ scanning area, which further attests to the smoothness and high crystalline quality of the film. These features are essential for reliable electrical performance.

**Figure 2:**
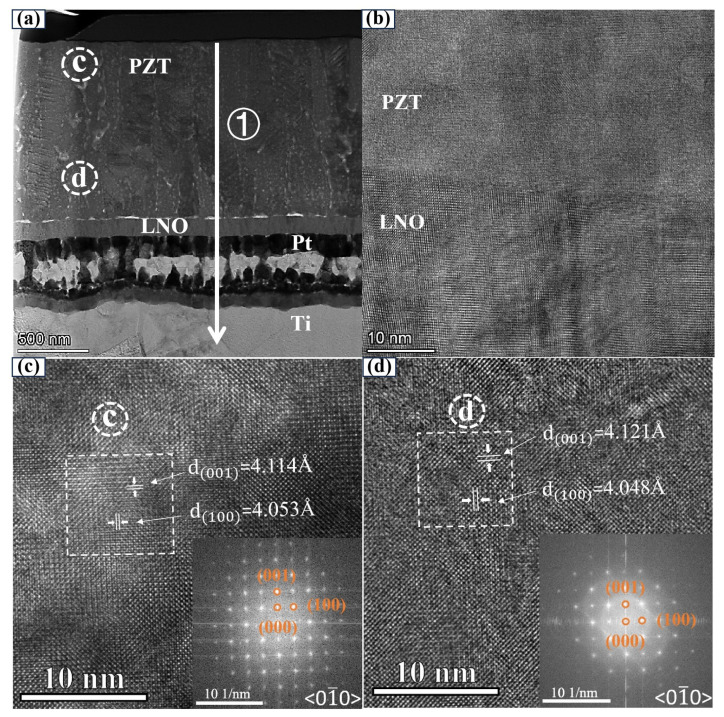
Nanoscale structural analysis: (**a**) cross-sectional bright field TEM image, mark ① and vertical arrow represent the region for line-scan EDS measurement in Figure 3, (**b**) HRTEM image of the PZT/LNO interface, (**c**,**d**) high-magnification lattice images with corresponding Fast Fourier Transformed Selected Area Diffraction Patterns (FFT-SAED, insets).

TEM analyses were performed to investigate the nanoscale structural and chemical composition characteristics. Figure 2a presents a low-magnification cross-sectional bright-field TEM image of the PZT/LNO/Pt/Ti heterostructure. No delamination is observed across the interfaces, suggesting good adhesion throughout the multilayer stack. The PZT film (~1.27 μm) exhibits a dense columnar microstructure free of macroscopic defects such as large pores or cracks. It does, however, show a small amount of nanopores (white dots in the image) resulting from the RTP process [24]. Because these pores are mostly isolated due to the short period of high-temperature exposure, they are not expected to deteriorate the electrical performance of the PZT film [24]. The PZT/LNO/Pt/Ti heterostructure shows clear variations in the microstructures of the constituent layers and interfaces. The Ti/Pt interface is rough and mechanically interlocked by a transition layer (the dark gray layer sandwiched between the Pt bottom electrode and the Ti substrate), which is ~100 nm thick and can be attributed to the thermal interdiffusion of the two metallic layers. Moreover, near the center of the Pt layer, which shows a deep dark gray contrast with a textured columnar grain morphology, there are embedded, semi-continuous light gray regions. These regions demonstrate the same contrast as that of the Ti substrate, indicating that they might be rich in thermally diffused Ti. The latter has been reported to occur at elevated temperatures through the grain boundaries of the Pt layer and subsequently oxidize inside it [12].

To further investigate the PZT/LNO interface at the nanoscale, high-resolution TEM (HRTEM) imaging was performed and the result is shown in Figure 2b. An atomically sharp boundary is observed between the two highly crystalline layers with no detectable secondary phases or amorphous “dead zone”, which are usually caused by interdiffusion. This confirms that the LNO layer acts not only as a crystallographic template, but also as a robust diffusion barrier, preventing the downward diffusion of Pb, Zr or Ti from the PZT film. Furthermore, to estimate the lattice distortion of the PZT film, high-magnification lattice imaging was performed on selected regions of Figure 2a, revealing a highly ordered atomic arrangement, as shown in Figure 2c,d. Fast Fourier Transform (FFT) analyses (*DigitalMicrograph software*, Version 3.52) were carried out for the square-encircled selected areas in Figure 2c,d, and the results are shown as insets. These FFT-SAED patterns display a high degree of crystallinity, especially for region c near the film surface, which shows single-crystal-like sharp diffraction spots (inset of Figure 2c). The measured lattice spacings demonstrate a tetragonal symmetry: the out-of-plane lattice parameters in Figure 2c,d are c = 4.114 Å and 4.121 Å, respectively, and the corresponding in-plane lattice parameters are a = 4.053 Å and 4.048 Å. These values match well with those from the standard ICDD PDF card (PDF#70-4060) for a tetragonal MPB PZT (Zr/Ti = 52/48) (a = 4.055 Å, c = 4.11 Å). The film strain ε*_f_* can be calculated using the equation below: [25]

ε*_f_* = (*a* − *a*_0_)/*a*_0_ (1)

where *a* represents the lattice parameter of the PZT film, *a*_0_ is the corresponding bulk lattice parameter. Calculations show that from the bottom region d to the surface region c, ε*_f_* in the out-of-plane direction decreases from 0.27% to 0.10%, while the compressive strain in the in-plane direction also decreases from −0.17% to −0.05%. This small negative in-plane strain is consistent with the well-matched CTEs between he PZT and the Ti substrate. In addition, the relaxation of the film strain from its bottom to the surface is well explained in the literature for thick films. Such a small in-plane compressive strain in the PZT film lays a crucial foundation for the optimization of its piezoelectric performance [26].

**Figure 3:**
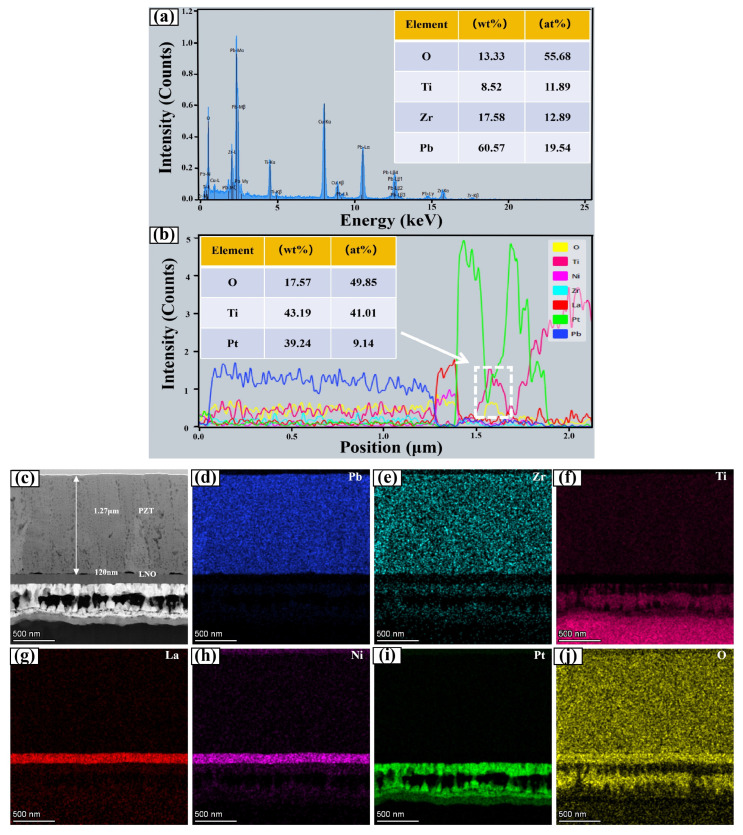
Nanoscale chemical composition analysis: (**a**) TEM EDS spectra with a quantitative analysis (inset), collected from region ① of Figure 2a, (**b**) EDS line-scan spectrum collected along the marked arrow direction in Figure 2a (inset: quantitative analysis of the Ti-rich region inside the Pt electrode layer), (**c**) cross-sectional HAADF-STEM image, and (**d**–**j**).

To quantitatively analyze the chemical composition of the PZT film, TEM-EDS point and line scans were performed. Figure 3a displays a representative EDS point-scan spectrum acquired from the middle bulk region of the PZT layer (labeled ① in Figure 2a). Only the elements O, Ti, Zr, and Pb were detected in Figure 3a (with no detectable signals from other elements), verifying the effective role of the LNO buffer layer as a diffusion barrier. A representative EDS point analysis reveals that the Zr/Ti atomic ratio is approximately 1.084, matching well with the theoretical value of 1.083 at the MPB. Additionally, the Pb/O atomic ratio is approximately 0.35, close to the 1:3 stoichiometric ratio (≈0.33), confirming the formation of a high-quality perovskite phase. The EDS line scan in Figure 3b, acquired along the white arrow in Figure 2a, shows the spatial elemental distribution across the PZT/LNO/Pt/Ti heterostructure. The PZT film occupies the position range from 0 to ~1.27 μm, where Pb, Zr, Ti, and O all show a relatively uniform and stable distribution, confirming its compositional stability. The LNO buffer layer is located in the position range from ~1.27 μm to ~1.4 μm, where the signals of La and Ni are mainly distributed. The position range between ~1.4 μm and ~1.9 μm corresponds to the Pt bottom electrode layer, where both strong Pt signals and noticeable Ti signals are observed. The latter shows two characteristic distribution zones. The first zone peaked in the middle of the Pt layer (at a position ~1.6 μm), decaying on both sides while being accompanied by an oxygen signal with a similar distribution. Such observations indicate diffusion of both elements into the Pt layer and a chemical reaction where they encounter each other. The second zone is located near the interface between the Pt layer and the Ti substrate, showing a decaying Ti signal as it penetrates into Pt. This zone corresponds to the transition layer observed in Figure 2a. Correspondingly, the Pt signal shows the opposite trend to that of the diffused-in Ti. It ramped up away from the first zone (“TiO_x_” zone), where it shows a minimum. The Pt signal reached a stable high value (as for bulk Pt) at the Pt/LNO interface before taking a nosedive to a noise level. On the other hand, heading towards the Ti substrate from the “TiO_x_” zone, the Pt signal first peaks at its bulk value and then slowly decayed down to zero through the Pt/Ti “transition layer” zone. Lastly, in the position range beyond ~1.9 μm, the Ti substrate is reached and displays a sole signal above the noise level, indicating successful suppression of oxygen penetration.

Moreover, High-Angle Annular Dark-Field (HAADF) imaging was performed on the cross-section of the PZT/LNO/Pt/Ti heterostructure, together with an EDS plane-scan of individual elements (Figure 3c–j). In Figure 3f–i, the interdiffusion of Pt and Ti is verified, while in Figure 3j, the oxidation of diffused Ti in the Pt layer is also validated. Meanwhile, the Pt/LNO interface is comparatively sharp and flat, with the LNO layer uniformly covering the Pt grains and serving as a conductive buffer for the PZT layer on top. It is noted that the diffusion of Pt towards the PZT film is effectively blocked by the LNO layer (Figure 3i) [11]. The latter shows chemically abrupt top and bottom boundaries, within which the La and Ni signals (Figure 3g,h) are strictly confined, revealing a ~120 nm thickness for this buffer layer. Consequently, a smooth and clean-cut LNO/PZT interface is achieved, consistent with the regular TEM result (Figure 2b). Additionally, the elemental maps for Pb, Zr, and Ti demonstrate a homogeneous distribution throughout the PZT layer without any signs of segregation or composition fluctuation (Figure 3d–f). They also all show a sharp depleted region where the LNO layer exists, reaffirming the role of a diffusion barrier that LNO played in addition to a crystallographic template. Together with the result shown in Figure 3b,i, it is concluded that corresponding EDS elemental plane-scan profiles of the PZT/LNO/Pt/Ti heterostructure show no discernible upward Pt migration into the PZT or downward diffusion of the Pb/Zr/Ti atoms. It is noted that, the Zr signal found in the Pt/Ti zone comes from the interfering signal of Mα for Pt (~2.048 keV), which significantly overlaps with Lα for Zr (~2.042 keV) [27]. Lastly, using quantitative EDS, the Ti/O atomic ratio in the “TiO_x_ zone” is estimated to be ~1:1.22, yielding a x value of ~1.22 (inset of Figure 3b). This observation indicates that the titanium oxide is sub-stoichiometric and hence conductive. Therefore, in the PZT/LNO/Pt/Ti heterostructure, not only is the oxidation of the Ti substrate suppressed, but the conductive electrode is also preserved with a pristine Pt/LNO interface (TiO_x_ is buried inside Pt). This is ideal for promoting an outstanding and robust piezoelectric performance in the (001)-textured PZT film.

With these nanoscale EDS results, the beneficial role played by the LaNiO_3_ layer in stabilizing the PZT/Pt and Pt/Ti interfaces during thermal processing can be interpreted from both thermodynamic and kinetic perspectives. Thermodynamically, because LNO shares the perovskite structure with PZT, the PZT/LNO interface exhibits lower interfacial energy than PZT grown directly on metallic electrodes. This structural compatibility reduces the nucleation barrier for the perovskite phase and promotes the formation of a dense, well-textured film. In addition, the mixed ionic–electronic conductivity of LNO allows partial equilibration of the oxygen chemical potential across the interface during thermal processing. Such equilibration reduces the thermodynamic driving force for oxygen transport toward the titanium substrate, thereby suppressing Ti substrate oxidation and mitigating interfacial reactions (forming sub-stoichiometric, conductive TiO_x_, instead of insulating TiO_2_, inside the Pt electrode layer). These effects collectively contribute to stabilizing the PZT/LNO/Pt/Ti heterostructure during rapid thermal crystallization in an O_2_ atmosphere. Such boosted stabilization can also be understood from a kinetic perspective. In polycrystalline thin films, oxygen transport is often dominated by grain-boundary and defect-assisted diffusion pathways. Direct growth of PZT on metallic electrodes like Pt typically produces interfaces with significant structural mismatch, which can introduce interfacial defects that act as fast diffusion channels. The introduction of a perovskite LNO buffer layer improves structural compatibility with PZT and promotes the formation of a dense film with fewer interfacial defects and grain-boundary pathways. Consequently, the effective oxygen diffusion toward the titanium substrate is reduced. This promoted growth of PZT on LNO also enables a rapid thermal crystallization via RTP, which further curtailed the supply of oxygen toward the Ti substrate. Moreover, due to this LNO-enabled, rapidly ramping, and short-duration RTP step, the out-diffusion of Ti was greatly mitigated and eventually stopped by the formation of TiO_x_ inside the Pt electrode layer.

Based on the EDS results and the above analysis, it can be inferred that during RTP in an oxygen atmosphere, the Pt grain boundaries are activated at elevated temperatures. This activation enables Ti atoms to diffuse along these intergranular pathways [12,28,29], and react with the diffused-in oxygen. Consequently, sub-stoichiometric titanium oxide (TiO_x_) grows in situ in a semi-continuous fashion (expanding laterally and vertically from the grain boundaries of Pt), until it becomes self-limited due to a lack of reactant supply. Located in the middle of the Pt layer and acting as a conductor itself [28,30], the resulting TiO_x_ “band” acts as a sink for O and a diffusion barrier for Ti without ruining the bottom electrode. Not only does it protect the Ti substrate by preventing further penetration of oxygen, but it also keeps the metallic Ti within the Pt electrode, hence maintaining the chemical stoichiometry of the MPB PZT film (Zr/Ti = 52/48). The activation of the intergranular diffusion of Ti and the subsequent reaction need a high processing temperature, which is supported by our previous work on the low-temperature sputter-deposition (@450 °C) of BiFeO_3_ films onto Ti substrates [16]. In this work, no detectable amount of diffused Ti was revealed in the Pt bottom electrode layer. Therefore, the rapidly ramping, short-duration RTP process, enabled by the introduction of the LNO buffer layer, is not only a crystallization step for PZT with a reduced thermal budget but also a thermal treatment leading to the formation of a diffusion sink/barrier buried inside the Pt electrode layer. The resulting dual-buffer and dual-barrier configuration—where the LNO layer acts as the orientation-defining template and final diffusion-shield [11], while the Pt layer induces the (100) LNO template growth [31], and a self-limiting oxidative reaction leads to the formation of a diffusion barrier/sink for Ti and O—is the key to preserve the chemical integrity of both the PZT and the Ti substrate, while at the same time allowing the formation of a desirable crystallographic orientation. Consequently, optimal ferroelectric and piezoelectric properties are expected for the PZT/LNO/Pt/Ti heterostructure.

**Figure 4:**
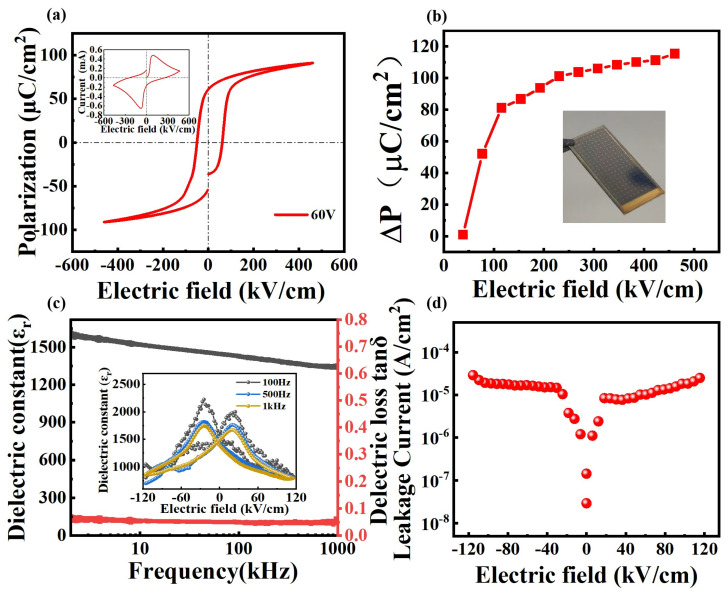
Electrical properties: (**a**) *P*−*E* hysteresis loop, (**b**) electric field-dependent pulsed polarization (Δ*P*) measurement results via PUND (inset is a picture of the sample for electrical characterizations), (**c**) frequency dependent dielectric constant and loss tangent (tan*δ*), with inset showing a typical ferroelectric *ε_r_*−*E* loop with a “butterfly” shape, (**d**) leakage current density (*J−E*) curve of the (001)-oriented PZT thick film on Ti. Solid squares in panel (**b**) and solid circles in panel (**d**) denote the measured experimental data.

The high degree of (001)-textured crystallinity revealed in the microstructural analysis translates directly into superior ferroelectric properties. Figure 4a displays the polarization–electric field (*P-E*) hysteresis loop of the PZT film measured at room temperature at a maximum electric field of 461 kV/cm. The film exhibits a well-saturated, square-shaped hysteresis loop with a large remnant polarization (*Pᵣ*) of ~61 μC/cm^2^ and a saturation polarization (*P_s_*) of ~91 μC/cm^2^, as well as a small coercive field (*E_c_*) of ~56 kV/cm. Such a large *Pᵣ*, one of the highest reported for PZT films integrated on metallic substrates, is a direct manifestation of the highly (001)-oriented crystalline structure, which allows for an efficient collective domain switching under an electric field applied along the film normal. The switching current curve (inset of Figure 4a) shows two sharp, symmetric peaks at the positive and negative coercive fields, confirming that the measured polarizations mostly stem from intrinsic ferroelectric polarization rather than leakage artifacts. To measure the intrinsic ferroelectric polarization of the PZT film, we performed PUND (Positive Up, Negative Down) pulse polarization measurements, which can eliminate the contribution to polarization charges from leakage current and linear dielectric capacitance. The PUND test results, i.e., the measured switchable polarization Δ*P* as a function of the applied electric field, are presented in Figure 4b. At the same maximum electric field as that of the *P-E* loop test (~461 kV/cm), Δ*P* is ~113 μC/cm^2^, which is nearly twice the remnant polarization *P*_r_ (~61 μC/cm^2^) from the *P-E* loop. This result confirms that the PZT film on Ti possesses outstanding ferroelectric properties. Moreover, Figure 4c presents the frequency-dependent dielectric property [32]. The film demonstrates a high relative dielectric constant (*εᵣ*) of ~1612 at 1 kHz and maintains a low dielectric loss (tan*δ*) below 0.06 over a broad frequency range of [1 kHz, 1 MHz] [33]. The characteristic “butterfly” shape of the *εᵣ*-*E* curve (inset of Figure 4c) is a hallmark of the film’s strong ferroelectric nature. Furthermore, the PZT film’s electrical integrity is confirmed by a low leakage current density of 2.1 × 10^−5^ A/cm^2^ at 120 kV/cm (Figure 4d), which is a result of its dense microstructure and chemical purity.

**Figure 5:**
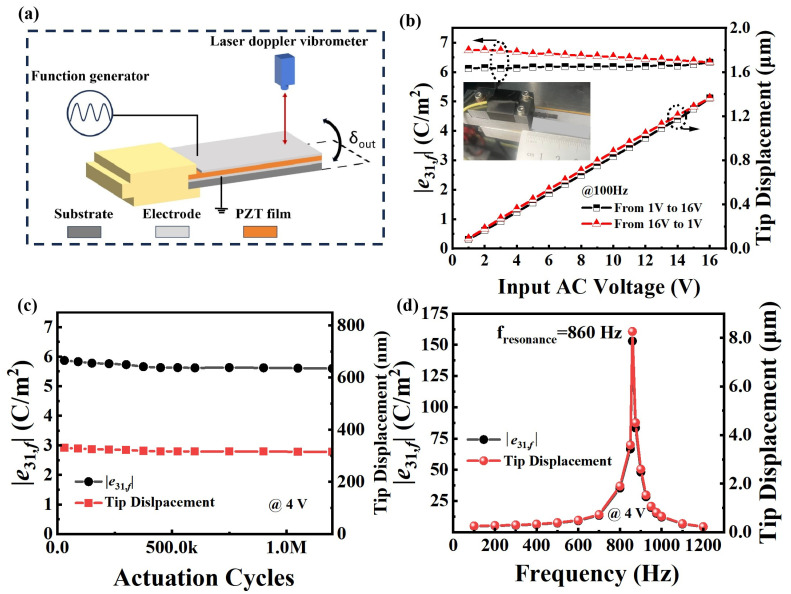
Transverse piezoelectric properties of the (001)-oriented PZT thick film on Ti: (**a**) schematic of the piezoelectric measurement setup, (**b**–**d**) tip displacement and corresponding transverse piezoelectric coefficient (∣*e*_31,*f*_∣) as functions of (**b**) amplitude of an input AC voltage (inset is a picture of the cantilever beam sample in the fixture for piezoelectric characterization), (**c**) the number of piezoelectric actuation cycles, and (**d**) the exciting frequency of the input AC voltage (@ a fixed AC voltage of 4 V).

Lastly, the magnitudes of the transverse piezoelectric coefficients (∣*e*_31,*f*_∣) of the PZT film were measured from the tip displacements of a cantilever beam (Figure 5a) diced from the Pt/PZT/LNO/Pt/Ti heterostructure. As shown in Figure 5b, the tip displacement increases almost linearly with increasing AC voltage, reaching ~1.36 μm at 16 V. The corresponding ∣*e*_31,*f*_∣ is maintained at a stable high value, ranging between 6.1 and 6.3 C/m^2^ when ramping up from 0 V to 16 V. It only increases slightly (from 6.3 to 6.7 C/m^2^) during ramping down (16V ⟶ 1V), which can be attributed to a fully poled polarization state [34]. This remarkable pseudo-linearity suggests that the piezoelectric response is dominated by the intrinsic piezoelectric effect, which is characteristic of a highly (001)-oriented PZT film [34,35,36]. Furthermore, δ and ∣*e*_31,*f*_∣ as functions of the piezoelectric actuation cycles are displayed in Figure 5c. After 1.2 × 10^6^ actuation cycles under an applied AC voltage of 6 V, the reductions in δ and ∣*e*_31,*f*_∣ are very small (~4%), indicating excellent fatigue resistance. The ac voltage used for the measurements presented in Figure 5b,c is at 100 Hz, far away from the resonance frequency of the cantilever beam. The latter is determined by a frequency sweep (Figure 5d) to be ~860 Hz. This is done to ensure quasi-static conditions for the measurement of *e*_31,*f*_.

Table 2 compares the key performance metrics of our PZT film against other commonly used ferroelectric films [6,10,16,37], i.e., PZT and BiFeO_3_ (BFO), on Ti substrates (there is no reported data for the integration of (K,Na)NbO_3_ films on Ti). For a rigorous comparison, the bottom electrode configuration, film thickness, preparation method, and polarization values at 100 kV/cm are also included in Table 2. Most notably, our PZT film achieves much higher polarization values (*P*_r_ ~ 61 μC/cm^2^) than its PZT peers, together with a much larger dielectric constant (*ε_r_* ~ 1612). Based on the positive correlations between these two parameters and the transverse piezoelectric coefficient *e*_31,*f*_ [34,38], a superior *e*_31,*f*_ coefficient was expected and that is what was revealed experimentally. ∣*e*_31,*f*_∣ is ~6.7 C/m^2^ in a fully poled state, and ~6.1 C/m^2^ for the unpoled state. This transverse piezoelectric property significantly outperforms that of its PZT peers and the lead-free counterpart of BiFeO_3_. This performance boost can be attributed to the synergistic effect of the following three factors:

1. The LNO buffer layer, which ensures a high (001) orientation with an optimal polar axis alignment, and at the same time acts as a diffusion barrier for the PZT and the Pt electrode.
2. The “rapidly ramping, short duration” RTP process, which yields a dense, highly crystalline microstructure essential for electrical and piezoelectric performance, while at the same time limiting the inter-layer diffusion and ensures the chemical integrity of both the PZT film and the highly reactive Ti substrate.
3. The matching CTEs between the Ti substrate and the PZT film, which helps to minimize the film’s residual stress. A slightly compressive residual stress is beneficial to maintaining a high electric polarization, providing a crucial foundation for the electrical and piezoelectric performance of a ferroelectric film.

## 4. Conclusions

In summary, high-quality, (001)-oriented PZT thick films (~1.27 μm) were successfully integrated onto Ti substrates, via a two-step process combining low-temperature RF sputter-deposition (@400 °C) with a RTP. The introduction of a conductive LNO growth template proves critical in lowering the nucleation energy barrier for the (001) perovskite PZT. Moreover, the LNO layer acts synergistically with the RTP to suppress substrate oxidation and mitigate interfacial diffusion and chemical reactions. Consequently, the resulting (001)-textured PZT film exhibits a dense columnar grain microstructure, and delivers outstanding electrical and piezoelectric performance. A large remnant polarization of ~61 μC/cm^2^, a low leakage current (J ≈ 2.1 × 10^−5^ A/cm^2^ @ 120 kV/cm), and a high dielectric constant (*ε_r_* ~ 1612 @ 1 kHz) are demonstrated by the PZT film. Most notably, a large transverse piezoelectric coefficient *e*_31,*f*_ of ~−6.7 C/m^2^ is achieved, significantly outperforming reported piezoelectric films grown on Ti. This work effectively resolves the thermal budget conflict between achieving a high degree of crystallinity in the PZT film and avoiding the deterioration of devices on reactive metal substrates, thereby establishing a scalable and cost-effective pathway for flexible piezo-MEMS devices.

## Figures and Tables

**Table 1:** Process parameters for the sputter-deposition of PZT/LNO/Pt/Ti heterostructures.

Layers	Base Pressure (Pa)	Target–Substrate Distance (mm)	Target Diameter (Inches)	Substrate Temperature (◦C)	Sputtering Pressure (Pa)	Sputtering Power (W)	Sputtering Atmosphere	Targeted Thickness (nm)
**Ti (adhesion)**	2.0 × 10^−4^	50	3	300	0.3	55	Ar	10
**Pt**								300
**LaNiO_3_**				400		100	Ar/O_2_~4:1	120
**Pb(Zr,Ti)O_3_**					1.2	130		1300

**Table 2:** Piezoelectric performances of sputtered PZT and BiFeO_3_ (BFO) films on Ti.

Source	This Work	[6]	[10]	[37]	[16] (BFO)
*Bottom electrode*	Ti/Pt	Pt	—	LaNiO_3_	Pt
*Film thickness* (μm)	1.27	3.8	0.5	0.6	0.8
*Preparation method*	RF magnetron sputtering (400 °C) +RTP (2.5 min 640 °C)	RF magnetron sputtering (600 °C) + furnace annealing (60 min 650 °C)	RF magnetron sputtering (300 °C) + furnace annealing (30 min 650 °C)	sol–gel + furnace annealing (650 °C)	RF magnetron sputtering (450 °C)
$E_{c}\;(kV/cm)$	56	70	45	—	145
$E_{b}\;(kV/cm)$	850	—	—	—	—
$P_{s}\;(\mu C/cm^{2})@100\;kV/cm$	62	30	0.67	—	72
$P_{s}\;(\mu C/cm^{2})$	91	35	—	27	84
$P_{r}\;(\mu C/cm^{2})$	61	20	0.5	—	72
$\varepsilon_{r}$	1612	506	120	534	273
${Converse\;e}_{31,f}$ $(C/m^{2})$	6.1–6.7	3.6–4.3	—	—	2.2

## Data Availability

The original contributions presented in this study are included in the article. Further inquiries can be directed to the corresponding authors.
